# Efficacy and Safety of Micro‐Plasma Radiofrequency Combined With Subcision for Acne‐Induced Atrophic Scars: An Exploratory Case Series

**DOI:** 10.1111/jocd.71200

**Published:** 2026-09-28

**Authors:** Gendi Li, Fuhai Han, Mengdan Wen, Yi Huang, Shuxia Long, Ci Ren, Yongpeng Su

**Affiliations:** ^1^ Department of Medical Aesthetics The First People's Hospital of Qinzhou Qinzhou GuangXi China; ^2^ Department of Dermatology The First People's Hospital of Qinzhou Qinzhou GuangXi China; ^3^ Department of Dermatologic Surgery Shenyang Seventh People's Hospital Shenyang Liaoning China

**Keywords:** acne‐induced atrophic scars, correlation analysis, efficacy, Fitzpatrick skin type III, hyperpigmentation, micro‐plasma radiofrequency, safety, subcision, treatment outcomes

## Abstract

**Background:**

Post‐inflammatory hyperpigmentation (PIH) represents a major concern in the treatment of acne‐induced atrophic scars, particularly among individuals with intermediate to dark skin phototypes (Fitzpatrick skin types III–V). Although micro‐plasma radiofrequency (MPR) is theoretically associated with a lower risk of PIH, evidence regarding its use in type III skin remains limited.

**Objective:**

To preliminarily evaluate the efficacy and safety of combined MPR and subcision in patients with Fitzpatrick skin type III atrophic acne scars, and to explore correlations among baseline characteristics, treatment variables, and postoperative outcomes.

**Methods:**

We retrospectively analyzed patients with acne‐induced atrophic scars treated at our hospital between 2017 and 2024. Comprehensive clinical data, including demographic information, scar characteristics, treatment protocols, and posttreatment outcomes, were systematically collected. Treatment efficacy was evaluated by assessing improvements in scar appearance, while safety was monitored by documenting any adverse effects associated with the procedure.

**Results:**

Twelve patients (mean age 25.2 ± 4.1 years) completed three treatment sessions. Crust separation occurred within 15 days in all cases, with erythema resolving within 60 days in 91.7%. The overall complication rate was 8.3% (one transient hyperpigmentation). By ECCA assessment, no patient achieved “Cure” (0%); 58.3% were “Markedly effective”, 33.3% “Effective” and 8.3% “Ineffective”, yielding a total effective rate of 91.6%. Patient satisfaction (GAIS) revealed 25% “very satisfied”, 50% “satisfied”, and 25% “less satisfied”, with an overall satisfaction rate of 75%. Notably, a discordance was observed between objective efficacy and subjective satisfaction.

**Conclusions:**

This combination regimen showed preliminary evidence of favorable efficacy with a manageable PIH risk in type III patients, findings that warrant validation in larger, adequately powered cohorts in darker skin phototypes. The observed discordance between 91.6% objective efficacy and 75% satisfaction underscores the necessity of dual‐endpoint assessment and expectation management in aesthetic scar treatment. Exploratory correlation analyses suggested potential associations between baseline demographic features, anesthesia selection, and treatment outcomes. These preliminary findings, if validated, may inform individualized treatment planning.

## 1 Introduction

Atrophic acne scars arise from dermal damage caused by acne inflammation, resulting in permanent tissue loss upon healing [1]. In ethnic skin, preventing post‐inflammatory hyperpigmentation (PIH) is a primary goal when treating acne [2]. The same principle applies to acne scar treatment: selecting a modality with low PIH risk is particularly crucial for individuals with skin of color. PIH represents a particular concern in individuals with intermediate to dark skin phototypes (Fitzpatrick skin types III–V) [3]. Although MPR is theoretically associated with a lower risk of PIH compared to conventional laser therapies [4], existing evidence has primarily focused on skin types II–III, with specific efficacy data for type III as an intermediate phenotype remaining scarce. While direct comparative data between monotherapy and combination therapy are lacking, the rationale for combining MPR with subcision is based on their complementary mechanisms at different anatomical depths, as supported by a prior uncontrolled study [5].

The outpatient population at our institution predominantly exhibits Fitzpatrick skin type III, with types IV–V representing a smaller proportion. Given that type III skin demonstrates intermediate chromophore reactivity between lighter and darker skin phototypes, it may serve as a critical bridge population for evaluating the safety of MPR prior to extending its application to darker skin types. Therefore, this study retrospectively analyzed patients with Fitzpatrick skin type III atrophic acne scars who received combined MPR and subcision between 2017 and 2024, aiming to evaluate the efficacy and safety of this combined regimen in such intermediate phenotypes and to establish a clinical foundation for its subsequent application in populations with darker skin phototypes. This study also conducted exploratory correlation analysis to explore associations between baseline data, anesthesia methods, intraoperative pain, efficacy indicators, and adverse events, so as to provide a reference for optimizing clinical treatment strategies.

## 2 Materials and Methods

### 2.1 Participants

Between 2017 and 2024, a total of 14 patients with acne‐induced atrophic scars who underwent at least three sessions of combined MPR and subcision were identified in our department. After excluding 2 patients who concurrently received carbon dioxide fractional laser treatment, 12 patients were ultimately enrolled in this study. Among them, there were 11 women and one man. Ages ranged from 21 to 37 years, with a mean of 25.2 years. All patients were Fitzpatrick skin type III (*n* = 12). One patient developed new‐onset acne within the scarred area and received concomitant oral isotretinoin during the radiofrequency sessions, whereas the scars of all other patients were in a stable phase. Disease duration ranged from 3 to 10 years. Enrolled patients had no skin tumors, keloids, or systemic autoimmune diseases. Patients who were pregnant or lactating, those with implanted pacemakers or other foreign bodies, and those who had undergone any laser‐based treatment within the previous 6 months were excluded. All participants received MPR treatment for the first time. This study was conducted in accordance with the Declaration of Helsinki, and all patients provided written informed consent.

### 2.2 Equipment and Pharmaceuticals

- The Alma Plasma RF scar treatment system (Alma Lasers, Israel; 40.68 MHz radiofrequency‐induced microplasma; with both sliding and stationary modes) and a standard 0.4 mm × 12 mm hypodermic needle (RWLB; Guangxi Shuangjian Technology Co. Ltd., Nanning, China) were used.
- For anesthesia, compound lidocaine cream containing 2.5% lidocaine and 2.5% prilocaine (Beijing Ziguang Pharmaceutical Co. Ltd., Beijing, China) was applied. Additional anesthetic agents included 2% lidocaine injection with epinephrine (1:200000) for nerve block, and propofol, etomidate, and sufentanil citrate for intravenous general anesthesia.

### 2.3 Preoperative Preparation

The procedure was as follows: Before treatment, the patient cleansed the face, underwent photography, and lay in a supine position.

### 2.4 Anesthesia Methods

Based on patient preference, pain tolerance, and preoperative assessment, different anesthesia methods were administered.

- Surface anesthesia: Compound lidocaine cream was uniformly applied to the treatment area according to the distribution of acne scars, and the area was sealed with plastic wrap for 1.0 h.
- Nerve block anesthesia: Based on the distribution of acne scars, selective block of the infraorbital nerve, mental nerve, or supraorbital nerve was performed. A total of 5 mL of 2% lidocaine (containing epinephrine 1:200000) was injected at the corresponding nerve foramina or notches, with 0.5 mL administered at each site.
- Intravenous general anesthesia: Intravenous induction was performed by an anesthesiologist, with propofol 150 mg or etomidate 20 mg combined with sufentanil 10 μg; propofol was continuously infused via intravenous pump to maintain anesthesia during the procedure.

### 2.5 Therapeutic Technique

Rolling and superficial box‐shaped scars were targeted. At the edge of each scar, a 0.4 mm × 12 mm standard needle was inserted horizontally into the subcutaneous dermis, following the blunt blade subcision technique [6]. The needle was moved slowly and in parallel to separate fibrous bundles. Care was taken to control the needle's movement to prevent injury to the subcutaneous tissue and skin perforation. For larger and denser scars, the needle was inserted repeatedly at multiple angles to loosen the fibrotic tissue. During separation, an assistant applied pressure with gauze to control bleeding. After treatment, a gauze pad soaked in lidocaine injection was applied as a wet compress to the separated subcutaneous area before proceeding to the next step of treatment. The treatment head and parameters were set based on factors, such as the thickness of the skin at the treatment site and depth of the scar. All patients received dual treatment with MPR sliding and fixed‐point modes. First, a fixed treatment head was used for the fixed‐point treatment one to two times, at a power of 45–65 W. Subsequently, a roller treatment head was used for multidirectional sliding treatment three to five times, at a power of 50–70 W. For the areas around the eyes and mouth, the energy or number of treatments was reduced appropriately.

### 2.6 Postoperative Care

After treatment, the skin exhibited redness, swelling, and exudation, with occasional pinpoint bleeding and burning sensations. An ice pack was immediately applied to the treatment area until redness, swelling, and burning sensations subsided. A collagen dressing was placed between the ice pack and treatment area. Contact with water was avoided for 5–7 days. From the day of treatment, a medical‐grade recombinant human collagen functional dressing was applied three times a day until the skin healed and redness subsided. The treated areas were kept dry. Generally, scabs formed the day after treatment. Patients were instructed to allow the scabs to fall off naturally and not to remove them forcibly. They were advised not to wash or rub the treatment area and not to apply disinfectants or cosmetics without medical approval. Before the scabs fell, patients practiced physical sun protection. After the scabs fell off, the newly healed skin was kept moisturized, and sunscreen (SPF ≥ 30) was applied.

Additionally, patients avoided spicy or irritant foods, alcohol, and tobacco. Treatment frequency and cycle: ≥ 12 weeks per session, three sessions per treatment course. The treatment progress for each course was tracked and analyzed.

### 2.7 Outcome Measures

- Three months after three treatment sessions, the patient and their family evaluated subjective satisfaction with treatment outcomes using the 5‐point Global Aesthetic Improvement Scale (GAIS): 5 = very satisfied, 4 = satisfied, 3 = less satisfied, 2 = dissatisfied, 1 = worse. The satisfaction rate was calculated by dividing the number of satisfied cases by the total number of cases and multiplying by 100%.
- A professional dermatologist calculated the Efficacy Index (ECCA) by comparing patient images before and after three treatment sessions. Efficacy index calculation: (pretreatment lesion score − posttreatment lesion score)/(pretreatment lesion score) × 100%. 3 = Cure (index ≥ 90%), 2 = Markedly effective (60% ≤ index < 90%), 1 = Effective (30% ≤ index < 60%), 0 = Ineffective (index < 30%). Total effective rate = [(cured + markedly effective + effective) / total number] × 100%.
- Complications were recorded as follows: 0 = none; 1 = infection; 2 = skin necrosis; 3 = hyperpigmentation; 4 = hypopigmentation; 5 = hypertrophic scarring. The rate of complications was calculated by dividing the number of occurrences by the total number of cases and multiplying by 100%.
- Pain was assessed using a visual analog scale (VAS): 1 = no pain, no sensation; 2 = 1–3, mild pain; 3 = 4–6, moderate but tolerable pain; 4 = 7–10, severe pain.

### 2.8 Statistical Methods

Statistical analyses were performed using R version 4.3.2 (http://www.R‐project.org, The R Foundation) and Free Statistics software version 2.0. Descriptive statistics are presented as numbers and percentages of patients, or as mean values and standard deviations. The 95% confidence intervals for all binary outcomes were calculated using the Clopper‐Pearson exact method. Cohen's kappa coefficient was used to quantify the degree of inconsistency between objective efficacy (ECCA categories) and subjective satisfaction (GAIS scores), with corresponding *p* values and 95% confidence intervals reported. The association between physician‐assessed efficacy (ECCA grades) and patient‐reported satisfaction (GAIS scores) was evaluated using Spearman's rank correlation coefficient, as both variables are ordinal.

Pearson correlation coefficient was adopted to analyze the linear correlation among continuous variables including age, disease course, and Visual Analog Scale (VAS) pain score. A correlation matrix heatmap was plotted for visual presentation of correlation trends. The correlation coefficient *r* was interpreted as follows: |*r*| ≥ 0.7 indicated strong correlation, 0.4 ≤ |*r*| < 0.7 indicated moderate correlation, and 0.2 ≤ *|r*| < 0.4 indicated weak correlation. A two‐tailed *p* < 0.05 was considered statistically significant. For the Figure 2 correlation heatmap, Pearson's *r* was used for continuous variable pairs, while Spearman's *ρ* was used for any pairwise comparisons involving ordinal ECCA or GAIS scores.

## 3 Results

Twelve patients (one man and 11 women) with a mean age of 25.2 ± 4.1 years (range = 21–37 years) were enrolled (Table 1). Disease duration was ≤ 5 years in 75.0% of cases. The treatment areas in all patients included the bilateral cheeks. Of the 12 patients, 10 (83.3%) received topical anesthesia for all three sessions, 1 (8.3%) received nerve block anesthesia for all three sessions, and 1 (8.3%) received topical anesthesia for the first two sessions and intravenous general anesthesia for the third session due to pain intolerance. A minimum interval of 3 months was maintained between consecutive treatment sessions. Crust separation occurred within 15 days in all cases (100%). Erythema resolved within 60 days in 91.7% of patients. Three patients (25.0%) developed new acne lesions with subsequent scarring during the treatment course.

**TABLE 1:** Baseline characteristics of the study participants and Post‐treatment condition description.

Case	Sex	Age	Disease course	Anesthesia method	Crust detachment	Erythema resolution	Recurrence	Adverse reactions	Pain VAS scores	ECCA	GAIS scores
1	Female	22	4	Topical anesthesia	≤ 15 days	≤ 60 days	No	No	Moderate	Markedly effective	Satisfied
2	Female	24	5	Topical anesthesia	≤ 15 days	> 60 days	No	No	Moderate	Effective	Satisfied
3	Female	21	3	Topical anesthesia	≤ 15 days	≤ 60 days	No	No	Moderate	Markedly effective	Satisfied
4	Female	25	3	Topical anesthesia	≤ 15 days	≤ 60 days	No	No	Moderate	Markedly effective	Very satisfied
5	Female	24	5	Topical anesthesia	≤ 15 days	≤ 60 days	Yes	No	Moderate	Effective	Less satisfied
6	Female	23	4	Topical anesthesia	≤ 15 days	≤ 60 days	No	No	Moderate	Effective	Satisfied
7	Female	26	10	Topical anesthesia	≤ 15 days	≤ 60 days	No	No	Moderate	Ineffective	Less satisfied
8	Female	27	4	Intravenous anesthesia	≤ 15 days	≤ 60 days	Yes	No	No	Markedly effective	Satisfied
9	Female	25	9	Topical anesthesia	≤ 15 days	≤ 60 days	Yes	No	Severe	Markedly effective	Satisfied
10	Female	37	6	Topical anesthesia	≤ 15 days	≤ 60 days	No	Hyperpigmentation	Moderate	Effective	Very satisfied
11	Female	23	3	Topical anesthesia	≤ 15 days	≤ 60 days	No	No	Moderate	Markedly effective	Very satisfied
12	Male	26	4	Nerve block anesthesia	≤ 15 days	≤ 60 days	No	No	Mild	Markedly effective	Less satisfied

Postoperative complications (infection, necrosis, hyperpigmentation, hypopigmentation, and hypertrophic scarring) were recorded. Only one patient developed transient hyperpigmentation, resulting in an overall complication rate of 8.3% [95% CI: 0.0%–25.0%]. Intraoperative pain perception: one patient who received general anesthesia reported no sensation; one reported mild pain; nine described moderate but tolerable pain; and one reported severe pain. The mean pain VAS score was 2.8 ± 0.7. Among the 12 patients, 10 received topical anesthesia, while the remaining 2 received either intravenous anesthesia or nerve block anesthesia, which were combined as a non‐topical anesthesia group for subsequent correlation analyses.

No patient achieved a “Cure” rating (0%). Seven patients (58.3%) were rated as “Markedly effective,” four patients (33.3%) as “Effective,” and one patient (8.3%) was classified as “Ineffective” based on the ECCA assessment alone. The total effective rate (including “Cure,” “Markedly effective,” and “Effective”) was 91.6% [95% CI: 75.0%–100.0%], with a markedly effective rate of 58.3%. The mean efficacy index was 1.5 ± 0.7.

At the final follow‐up, 3 patients were “very satisfied,” 6 were “satisfied,” and 3 were “less satisfied”. Figure 1 presents the distribution of GAIS satisfaction scores across ECCA efficacy grades. No patients were dissatisfied or reported worsening. The overall satisfaction rate was 75% [95% CI: 50.0%–100.0%]. The mean GAIS patient satisfaction score was 2.0 ± 0.7. Representative cases demonstrating treatment response are presented in Figure 2.

**FIGURE 1:**
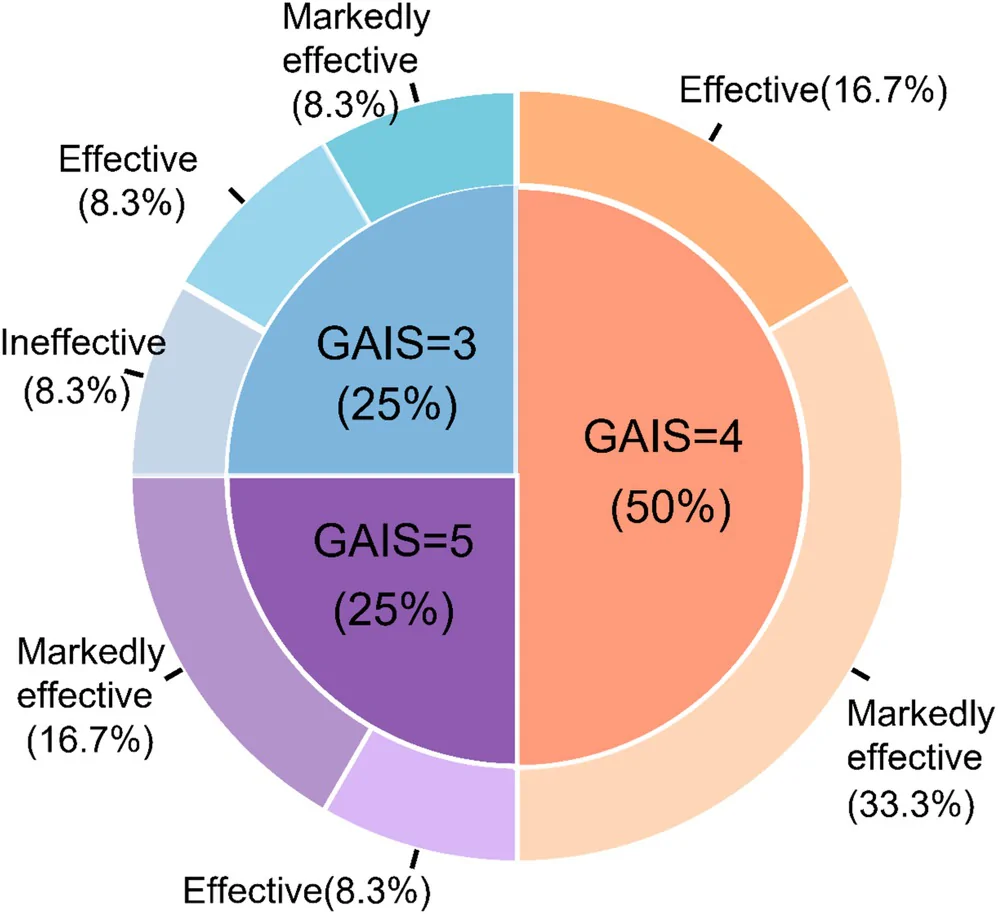
Association between patient satisfaction (GAIS score) and efficacy assessment (ECCA score). Patient satisfaction with the treatment of atrophic acne scars. 5 = very satisfied, 4 = satisfied, 3 = less satisfied, 2 = dissatisfied, 1 = worse. Dermatologist‐assessed efficacy of atrophic acne scar treatment. 3 = Cure; 2 = Markedly effective; 1 = Effective; 0 = Ineffective. The association between patient satisfaction (GAIS score) and efficacy assessment (ECCA score) was as follows: 25% reported being “very satisfied”, with 2 individuals (16.7%) corresponding to “markedly effective” and 1 individual (8.3%) to “effective”; 50% reported being “satisfied”, with 4 individuals (33.3%) corresponding to “markedly effective” and 2 individuals (16.7%) to “effective”; 25% expressed being “less satisfied”, with 1 individual (8.3%) corresponding to “markedly effective”, 1 individual (8.3%) to “effective”, and 1 individual (8.3%) to “ineffective”.

**FIGURE 2:**
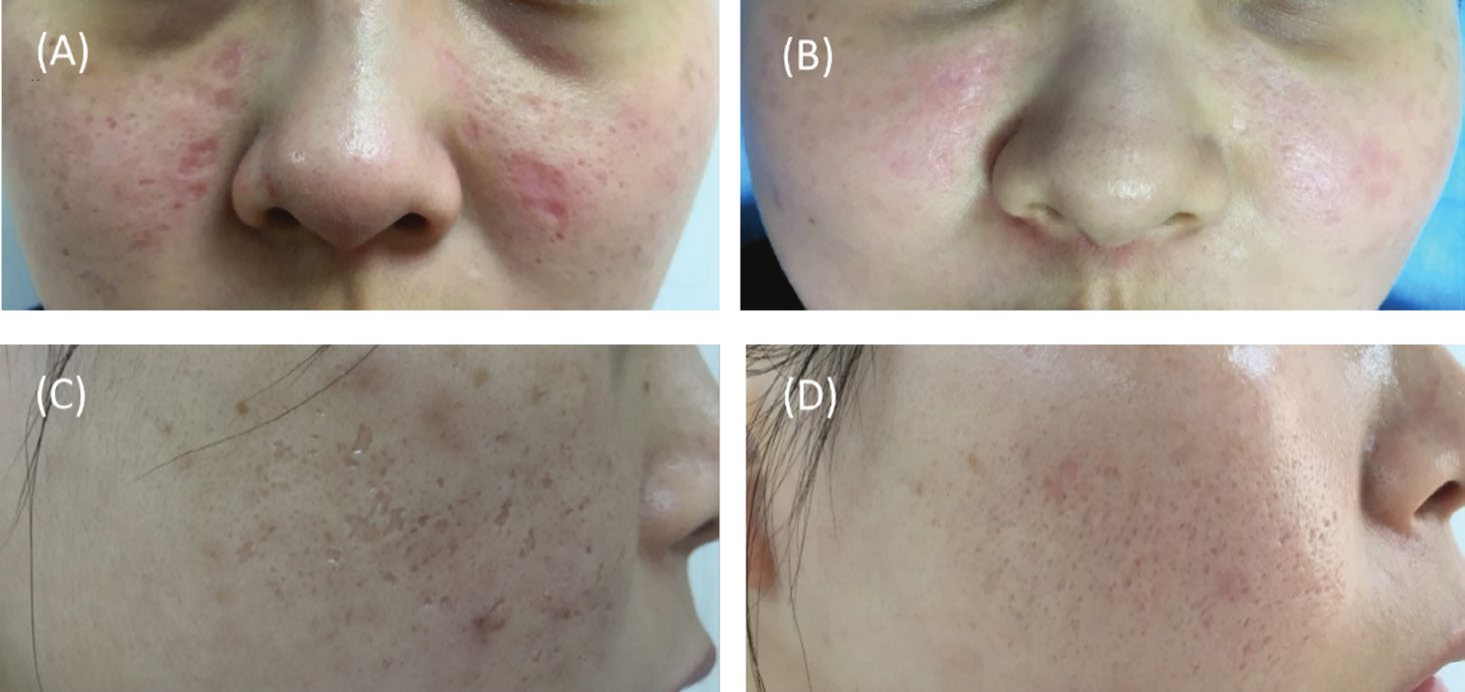
Presentation of Two Cases. Case 1: A 23‐year‐old woman presented with atrophic scars (predominantly rolling, with a few ice‐pick types) on both cheeks, which developed 3 years earlier following acne. Three sessions of MPR combined with subcision were performed. Pre‐ and posttreatment photographs are shown in (A, B). The ECCA improvement was 60%–90% (Markedly effective), and the patient rated her satisfaction as Grade 5 (very satisfied). Case 2: A 25‐year‐old woman presented with atrophic scars (chiefly boxcar and ice‐pick types) on both cheeks, which developed 3 years earlier after acne. Three sessions of the same combination therapy were performed. Photographs are shown in (C, D). The ECCA improvement was 60%–90% (Markedly effective), and satisfaction was again Grade 5 (very satisfied).

Cohen's Kappa analysis yielded Kappa = 0.43 (95% CI: −0.15 to 1.00, *p* = 0.12), with an overall agreement rate of 83.3% (10/12). Two out of 12 patients (16.7%) were objectively effective but subjectively dissatisfied. In addition, exploratory correlation analysis was performed to explore the associations between multiple study variables (Figure 3). Age exhibited a moderate positive correlation with disease course (*r* = 0.53). Disease course was moderately negatively correlated with ECCA score (Spearman's *ρ* = −0.45). Anesthesia method was strongly negatively correlated with VAS pain score (Spearman's *ρ* = −0.87, *p* < 0.001). Recurrence showed only a negligible negative correlation with VAS pain score (point‐biserial *r* = −0.14). Spearman's rank correlation analysis revealed a weak positive correlation between ECCA efficacy grades and GAIS satisfaction scores (Spearman's *ρ* = 0.37), though this association failed to reach statistical significance.

## 4 Discussion

Acne scars result from an abnormal wound‐healing response that causes dermal collagen loss. Atrophic scars—classified as ice‐pick, rolling, or boxcar subtypes—frequently involve fibrous tethering to the dermis [7, 8], necessitating subcision to release these bands before collagen remodeling procedures [9, 10]. Our two‐stage protocol combined subcision—which addresses deep fibrous septa with microplasma fractional radiofrequency (MPR) to stimulate superficial and middermal collagen remodeling. Subcision releases tethered scars while MPR induces neocollagenesis through electromagnetic radiation‐generated dermal heating [11, 12]. Unlike CO_2_ laser, MPR is chromophore‐independent, making it suitable for darker skin types [13]. The two techniques target complementary anatomical depths, and their sequential application may confer synergistic benefits.

The enrolled cohort presented relatively homogeneous baseline characteristics (Table 1), with all patients receiving three standard treatment cycles at adequate intervals, which facilitated comparison of efficacy and safety outcomes. Two patients receiving concurrent fractional CO_2_ laser therapy were excluded to maintain sample homogeneity, as the additive collagen remodeling effects would confound assessment of the MPR‐subcision combination.

Among the 12 enrolled patients, only one developed post‐inflammatory hyperpigmentation (PIH). Notably, this patient had concurrent active inflammatory acne and was receiving oral isotretinoin at the time of treatment. While isotretinoin use has traditionally raised concerns regarding impaired wound healing and hyperpigmentation, a recent systematic review indicated that this association is likely minimal [14]. In the present case, the PIH was more plausibly attributed to the active inflammatory acne itself, as cutaneous inflammation stimulates the release of inflammatory mediators—including prostaglandins, leukotrienes, and cytokines—that activate melanocytes and promote excess melanin deposition [15]. The PIH resolved spontaneously over 6 months without additional intervention, and the patient remained satisfied with the overall treatment outcome.

As shown in Figure 1, this study reveals a moderate but persistent discordance between objective morphological improvement and patient‐reported outcomes in atrophic acne scar therapy. While ECCA showed 91.6% treatment effectiveness, GAIS identified that only 25% of patients were “very satisfied” and 25% were “less satisfied.” This finding resonates with a growing recognition in cosmetic dermatology: structural improvement does not guarantee aesthetic satisfaction. Notably, the single ECCA “ineffective” case corresponded to “less satisfied” by GAIS, indicating convergence at the lowest efficacy extreme. However, the 16.6% gap between objective efficacy and overall satisfaction (91.6% vs. 75%) underscores that even patients with measurable clinical improvement may not perceive treatment as fully successful. Figure 1 presents the distribution of GAIS scores across ECCA efficacy grades. Despite 91.6% treatment effectiveness by ECCA, only 25% of patients reported being “very satisfied” by GAIS. Cohen's Kappa analysis showed fair‐to‐moderate agreement (Kappa = 0.43; 95% CI: −0.15 to 1.00; *p* = 0.12), with the non‐significant *p*‐value likely attributable to the small sample size. Pearson correlation analysis (Figure 3) revealed a strong negative correlation between anesthesia method and VAS pain score (*r* = −0.87), confirming its role in pain management. Weak correlational trends were observed between anesthesia method and postoperative recurrence (*r* = 0.26), as well as between anesthesia method and GAIS score (Spearman's *ρ* = −0.32). This weak GAIS association was analyzed via Spearman's rank correlation due to GAIS being an ordinal scale. Importantly, these weak associations cannot be interpreted as causal; patients receiving enhanced anesthesia typically had more severe baseline scars, and clinicians tended to apply higher energy under adequate analgesia, which may merely co‐occur rather than directly compromise dermal repair. Age and disease course were associated with poorer objective scar improvement, highlighting the need for intensified monitoring in older patients and those with longer disease duration. A weak positive correlation between ECCA and GAIS scores was detected via Spearman's rank correlation analysis (Spearman's *ρ* = 0.37), which suggests a trend toward internal consistency between objective and subjective evaluations, though the lack of statistical significance reinforces the observed evaluation deviation. Three factors may contribute to the discordance between objective improvement and subjective satisfaction. First, ECCA emphasizes scar depth and surface changes, whereas patients evaluate overall facial harmony and social confidence; subtle residual irregularities may disproportionately impact self‐perception. Second, no ECCA “cure” ratings (≥ 90% improvement) were achieved, with “markedly effective” (58.3%) predominating—in aesthetic medicine, patients often equate treatment success with invisible scars, a threshold this regimen did not reach. Third, the 25% “less satisfied” cohort included patients rated “markedly effective” by physicians, suggesting pre‐treatment counseling may inadequately convey realistic endpoints. These observations suggest satisfaction correlates more with alignment between expected and achieved appearance than with percentage improvement, though the small sample size warrants caution in interpretation.

Several limitations preclude definitive conclusions. First, the absence of control arms (standalone MPR, standalone subcision, and CO_2_ laser groups) prevents establishing whether the combination is superior to monotherapy or whether the proposed synergistic effect exists. Second, the small sample size (*n* = 12) with no a priori power calculation limits statistical robustness; post hoc analysis based on published CO_2_ laser data [16, 17] indicates approximately 48 subjects would be required to detect significant PIH differences (α = 0.05, power = 80%). Third, subgroup analyses by scar type, disease duration, or age could not be performed due to small cell frequencies, yielding unstable estimates. Fourth, the Kappa analysis showed only fair‐to‐moderate agreement, and the correlation between ECCA and GAIS did not reach statistical significance (*p* = 0.12), likely due to the small sample size. Fifth, the GAIS ceiling effect and exclusive Fitzpatrick type III cohort preclude generalization to darker phototypes (IV–V) (Figure 3).

**FIGURE 3:**
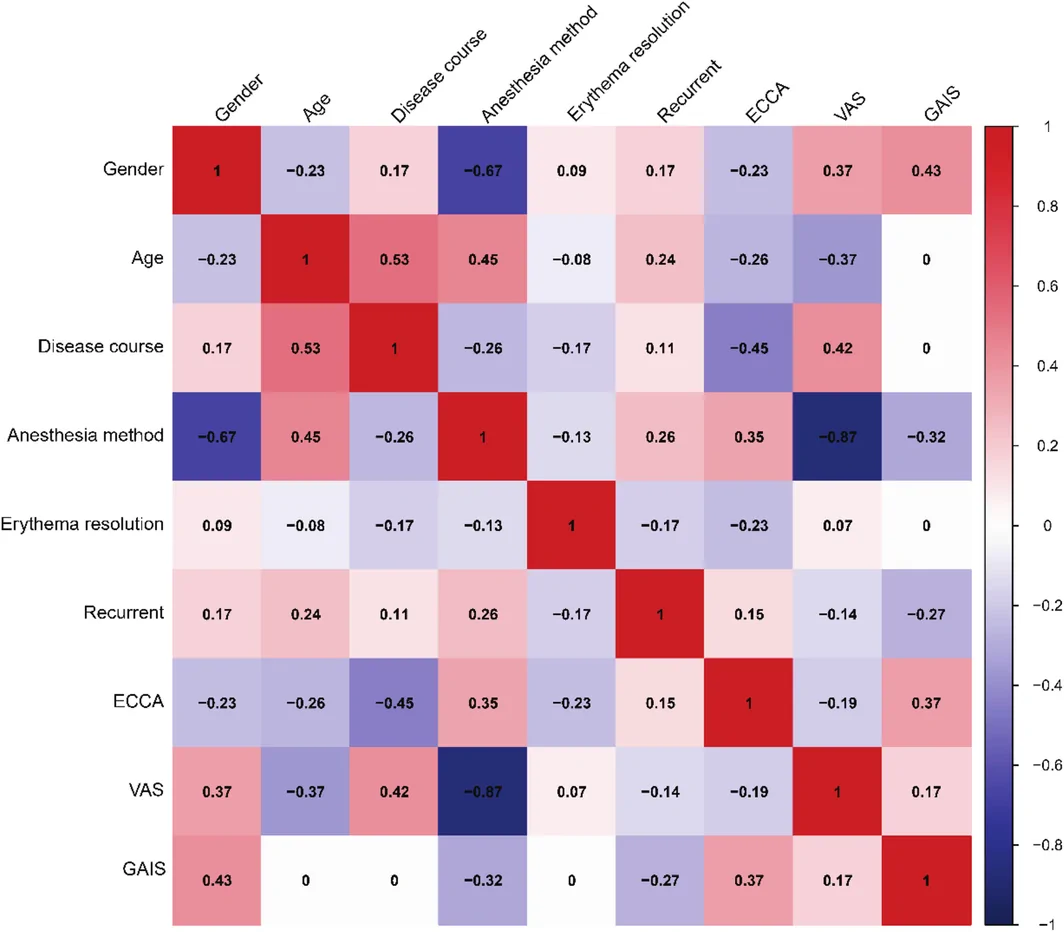
Correlation matrix heatmap of study variables. Pearson's correlation coefficients (*r*) are displayed for continuous variables; Spearman's rank correlation coefficients (Spearman's *ρ*) are used for pairwise associations between ordinal ECCA efficacy grades and GAIS satisfaction scores. Point‐biserial correlation coefficients (*r*) are used for pairs involving binary variables (recurrence) with continuous variables. Correlation coefficients and significance levels are presented. See Results section for detailed interpretation. Anesthesia method was dichotomized as topical anesthesia vs. non‐topical anesthesia (including intravenous and nerve block anesthesia) for correlation analysis. This correlation matrix heatmap showed linear relationships among patients' baseline profiles, treatment modalities, and postoperative outcomes. The main findings are presented below: 1. Correlations of baseline characteristics: Age exhibited a moderate positive correlation with disease course (*r* = 0.53), indicating that older patients tended to have longer disease duration of atrophic acne scars. Disease course was moderately negatively correlated with ECCA score (Spearman's *ρ* = −0.45), demonstrating that patients with longer disease course tended to have poorer objective scar improvement. 2. Correlations between anesthesia method and outcome indicators: Anesthesia method showed a strong negative correlation with intraoperative VAS pain score (Spearman's *ρ* = −0.87, *p* < 0.001). With the increase of analgesic intensity of anesthesia, patients' intraoperative pain score decreased significantly. Anesthesia method exhibited a weak positive correlation with postoperative recurrence (*r* = 0.26) and a weak negative correlation with GAIS satisfaction scores (Spearman's *ρ* = −0.32). 3. Correlations among efficacy and safety indicators: Recurrence showed a very weak negative correlation with VAS pain score (point‐biserial *r* = −0.14), suggesting a negligible linear trend between intraoperative pain perception and postoperative recurrence. A weak positive correlation between ECCA and GAIS scores was detected via Spearman's rank correlation analysis (Spearman's *ρ* = 0.37), which suggests a trend toward internal consistency between objective scar improvement and patients' subjective aesthetic evaluation, though this correlation was not statistically significant.

## 5 Conclusion

This combination regimen showed favorable efficacy with a low incidence of PIH in type III patients, providing preliminary evidence that warrants validation in larger, adequately powered cohorts. However, achieving “effective” ratings by physician assessment represents only partial success in aesthetic acne scar care. Optimal treatment optimization requires simultaneous pursuit of objective improvement and patient‐perceived benefit. This study advocates for integrated outcome measurement in cosmetic dermatology trials and highlights the value of managing expectations as meticulously as managing scars. Correlation analysis suggested that baseline age, disease course, and anesthesia method may be associated with intraoperative pain, adverse reactions, recurrence, and therapeutic effects. However, due to the small sample size and exploratory nature of these analyses, these findings should be considered hypothesis‐generating rather than confirmatory. If validated in future studies, individualized anesthesia selection and targeted interventions might enhance the overall benefits of combination therapy.

## Author Contributions

Gendi Li, Mengdan Wen and Shuxia Long: Curation; Gendi Li and Fuhai Han: Formal analysis; Gendi Li, Yongpeng Su and Yi Huang: Investigation; Gendi Li. and Ci Ren: Methodology; Gendi Li: Writing – original draft; Ci Ren and Yongpeng Su: Writing – review and editing. All authors have read and agreed to the published version of the manuscript. Yongpeng Su and Ci Ren are Co‐corresponding authors of the article.

## Funding

Guangxi Zhuang Autonomous Region Health Commission Self‐funded Research Project (Grant No. Z20200037).

## Ethics Statement

This study was approved by the Ethics Committee of the First People's Hospital of Qinzhou City (approval number A‐20251101). It was conducted in accordance with the Declaration of Helsinki, and all patients provided written informed consent.

## Consent

All participants provided informed consent.

## Conflicts of Interest

The authors declare no conflicts of interest.

## Data Availability

The data that support the findings of this study are available from the corresponding author upon reasonable request.
